# Square technique—A treatment for cellulite with large size particle hyaluronic acid

**DOI:** 10.1002/ski2.290

**Published:** 2023-09-23

**Authors:** Luciana de Matos Lourenço, Lara Apendino Colla, Maria Gabriela Ortiz de Noronha, Thomaz Rezende Izzo, Rosa Sigrist

**Affiliations:** ^1^ Private Practice in Dermatology São Paulo Brazil; ^2^ Hospital das Clínicas da Faculdade de Medicina da USP Universidade de São Paulo São Paulo Brazil

## Abstract

Gynoid lipodystrophy, also called cellulite, is defined as a metabolic disorder of the subcutaneous tissue that alters body contour. It is rare in men but affects 80%–90% of adult women.The authors describe a technique for the treatment of cellulite with non‐traumatic subcision^TM^ of septa by cannula associated with large‐gel‐particle hyaluronic acid (HA). It uses a special marking, a safe plan and a specific product. The product being used is Subskin Aeskins, in a subcutaneous plan as no major blood vessels were found.The authors report good aesthetic results with the proposed technique. The patients reported a high degree of satisfaction.The product chosen has high G prime and large particles, which accounts for resistance to deformation and a high lifting power. Moreover, HA is a biocompatible, absorbable product and considered a safe filler. This technique allows the treatment of an area with various surface irregularities.The Square Technique proved to be a minimally invasive technique, with fast results, no major risks and little downtime, bringing satisfaction to patients and improving their self‐confidence and quality of life.

## Graphical abstract

Our article presents a technique for the treatment of gynoid lipodystrophy, commonly known as cellulite, utilising non‐traumatic cannulated treatment of the septa with large‐gel‐particle hyaluronic acid (HA). The technique, employing a special marking, safe plan, and the use of HA with high G prime and large particles, yielded good aesthetic results and high patient satisfaction. With its resistance to deformation and high lifting power, HA proved to be a biocompatible and safe filler, enabling the treatment of surface irregularities. The Square Technique showcased its efficacy as a minimally invasive approach, delivering fast results, minimal risks, and enhancing patients' self‐confidence and quality of life.

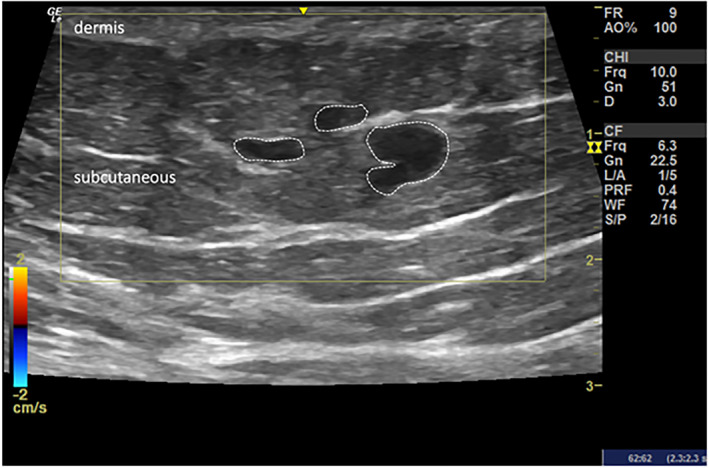

## 1 INTRODUCTION

Gynoid lipodystrophy, also called cellulite, is defined as a metabolic disorder of the subcutaneous tissue that alters body contour leading to an irregular appearance of the skin called “orange peel”.^1^, ^2^, ^3^ It is rare in men but affects 80%–90% of adult women and is mainly located on the buttocks and thigh.^1^, ^2^, ^3^

## 2 PATHOPHYSIOLOGY

Cellulite is multifactorial. The outstanding factors include connective tissue structure, oestrogen action, endothelial dysfunction and genetic predispositions. It is also considered inflammatory and biochemical alterations in the subcutaneous tissue.^1^, ^2^, ^3^, ^4^, ^5^

As a result of the endocrine‐metabolic microcirculatory disorder, it might cause interstitial matrix alterations and structural changes in the subcutaneous adipose tissue.^1^, ^2^ Changes occurring in the course of cellulite formation are fibrosis and sclerosis, that manifest clinically by topographic disorders of subcutaneous tissue as a wavy skin surface with multiple nodules and depressed areas, oedema and abnormal fibrosis.^1^, ^2^, ^3^

## 3 ANATOMY OF FIBROUS SEPTA

Magnetic resonance imaging studies have confirmed that cellulite depressions are associated with a significant increase in the presence and thickness of underlying subcutaneous fibrous septa. These fibrous septa segregate fat into channels. As the fat layer expands, it is projected superficially, creating an irregular appearance of the skin. Females with cellulite have a greater percentage of perpendicular septa compared with males or females without cellulite.^3^, ^4^, ^6^ A study with fresh frozen specimen showed that the mean number of subdermal fat lobules was significantly higher in men and they have a greater number of septal connections per measured areas in the superficial fatty layer. Female body has a significantly greater fat lobule width and height in this layer.^7^

There is greater tension on these fibrous septa from standing, pinching or active muscle contraction (due to communication with the underlying musculoaponeurotic system), which can worsen their clinical appearance.^4^

## 4 CELLULITE SEVERITY RATING SCALES

The point Nürnberger–Muller (NM) scale was used to classify cellulite for decades. This categorises skin appearance into 4 grades (0‐III): I‐ visible changes with skin clamping or muscle contraction; II‐ visible changes without manipulation; and III‐ visible changes associated with nodules.^4^, ^6^, ^8^, ^9^

The Cellulite Severity Scale by Hexsel et al. has become the new standard classification system for clinical evaluation and treatment response.^9^, ^10^ This scale adds four additional clinical morphologic features to the NM scale: the number of evident depressions, the depth of the depressions, the morphological appearance of skin surface alterations and the grade of laxity. The severity of each item is graded from 0 to 3. The sum could be 0 in case of complete absence.^9^

The clinical assessment of cellulite remains subjective despite the clear qualitative and quantitative measures taken to make evaluation more effective. There are many different treatments which are all designed to minimise the cellulite appearance and to achieve a smoother skin surface.^4^, ^6^, ^8^, ^9^, ^11^

## 5 TREATMENTS

### 5.1 Topical treatment

Some actives are used in topical products to stimulate circulation, lymphatic drainage and local lipolysis. Some of the most common actives are caffeine, vitamin A and botanical extracts. All with limited evidence in the treatment of cellulite.^3^, ^4^, ^5^, ^6^, ^12^

### 5.2 Extracorporeal shock wave therapy

Extracorporeal shock‐wave therapy demonstrated improvement in microcirculation, neocollagenesis and lymphatic drainage. There is also an additional benefit for patients in reducing the cellulite appearance and improving the skin firmness.^1^, ^4^, ^8^, ^12^, ^13^

### 5.3 Laser

Lasers which emit energy to the dermis/subcutaneous plane can stimulate collagen remodelling and increase microcirculation by heating the local tissue. The impact of these devices is not very substantial in terms of adipolysis or even disruption of the fibrous septa that characterise cellulite, but they can improve the appearance of the skin and smooth the surface. The main laser technology that has been proven effective to treat cellulite is 1440 nm Nd:YAG laser.^6^, ^8^, ^12^, ^13^

### 5.4 Radiofrequency

Radiofrequency technology has become a good treatment in aesthetic medicine with many indications due to its versatility, efficacy, and safety. Radiofrequency devices deliver thermal energy to the dermal and subcutaneous plan by elevating the tissue temperature, collagen denaturation, remodelling and leading to neocollagenesis. The lipolysis is also triggered. Studies demonstrated a significant improvement in cellulite appearance.^1^, ^5^, ^8^, ^12^, ^13^

### 5.5 Subcision^TM^

Manual subcision^TM^ of cellulite was first performed by Hexsel. The release of the fibrous septations alleviates the traction exerted on the skin causing the redistribution of tension between the fat lobules into the new spaces created to produce a smoother appearance to the skin. Besides that, it promotes the formation of new connective tissue through “autologous filling” from the bruises that are produced.^3^, ^5^, ^10^, ^12^, ^13^, ^14^

Traditionally, subcision^TM^ has been performed manually, using a 16 or 18 gauge needle inserted in the deep dermis to release the fibrous septations under local anaesthesia. It is recommended for cellulite depressions present at rest, not for depressions visible only with muscle contraction. Depressions are marked immediately pre‐procedural with the patient standing in a relaxed position.^3^, ^5^, ^10^, ^12^, ^13^, ^14^

Avoidance of strenuous physical activity for 1–2 weeks and use of a compressive garment for 2–4 weeks are recommended following any type of subcision^TM^. Subcision^TM^ can be divided into manual, vacuum‐assisted, and laser‐assisted methods. Manual subcision^TM^ is dependent on the skill and technique of the practitioner.^3^, ^5^, ^10^, ^12^, ^13^, ^14^

### 5.6 Chemical subcision

#### 5.6.1 Collagenase clostridium histolyticum

Collagenase clostridium histolyticum (CCH) is composed of 2 collagenases (AUX‐I and AUX‐II) that hydrolyse collagen under physiologic conditions, resulting in disruption of collagen structures (e.g., fibrous septae).

In a randomized, double‐blind study, which uses Injections of CCH 0.84 mg administered in women every 3 weeks over a 43‐day period significantly improved moderate to severe cellulite appearance versus placebo.

Overall, CCH treatment was well tolerated, with a low rate of patient discontinuation.^15^, ^16^, ^17^

#### 5.6.2 Injectable collagen biostimulators and dermal filler

Another up‐to‐date option to treat cellulite is the new generation dermal fillers injections, such as calcium hydroxyapatite (CaHa) and poly‐l‐lactic acid (PLLA). These fillers have been used to treat scars and can also be applied to smoothen the cellulite‐induced skin irregularities. Coleman and Pozner described various ways to combine treatments from their clinical experience to treat cellulite. They recommend combining subcision^TM^ or subdermal technologies, with PLLA or HA filler in patients with cellulite and volume loss.^5^, ^13^

The studies available in the literature showed better success of combined treatments than a single treatment. As cellulitis is a multifactorial disease, it requires a multidisciplinary approach to treatment.^5^, ^12^

## 6 MATERIALS AND METHODS

Authors describe a technique for the treatment of cellulite that uses a special marking, performing subcision^TM^ of septa by cannula in a safe plane with a specific product.

Twelve patients underwent this procedure. The ideal pacient for this technique is the patient without skin flaccidity, with atrophic lesions and surface irregularities.

To determine the specific site for application, the authors used the areas with the greatest number of lesions, they propose the treatment of an area with various surface irregularities. Depressions are marked immediately pre‐procedural with the patient standing in a relaxed position.

The product chosen for this technique was a large particles HA gel (800–1800 μm), with high G prime, filled in 10 and 20 mL syringes. The product used by the authors was Sofiderm SubSkin^®^.

The cannula used was the 18G × 70 mm. The syringe used was 3 mL.

**FIGURE 1:**
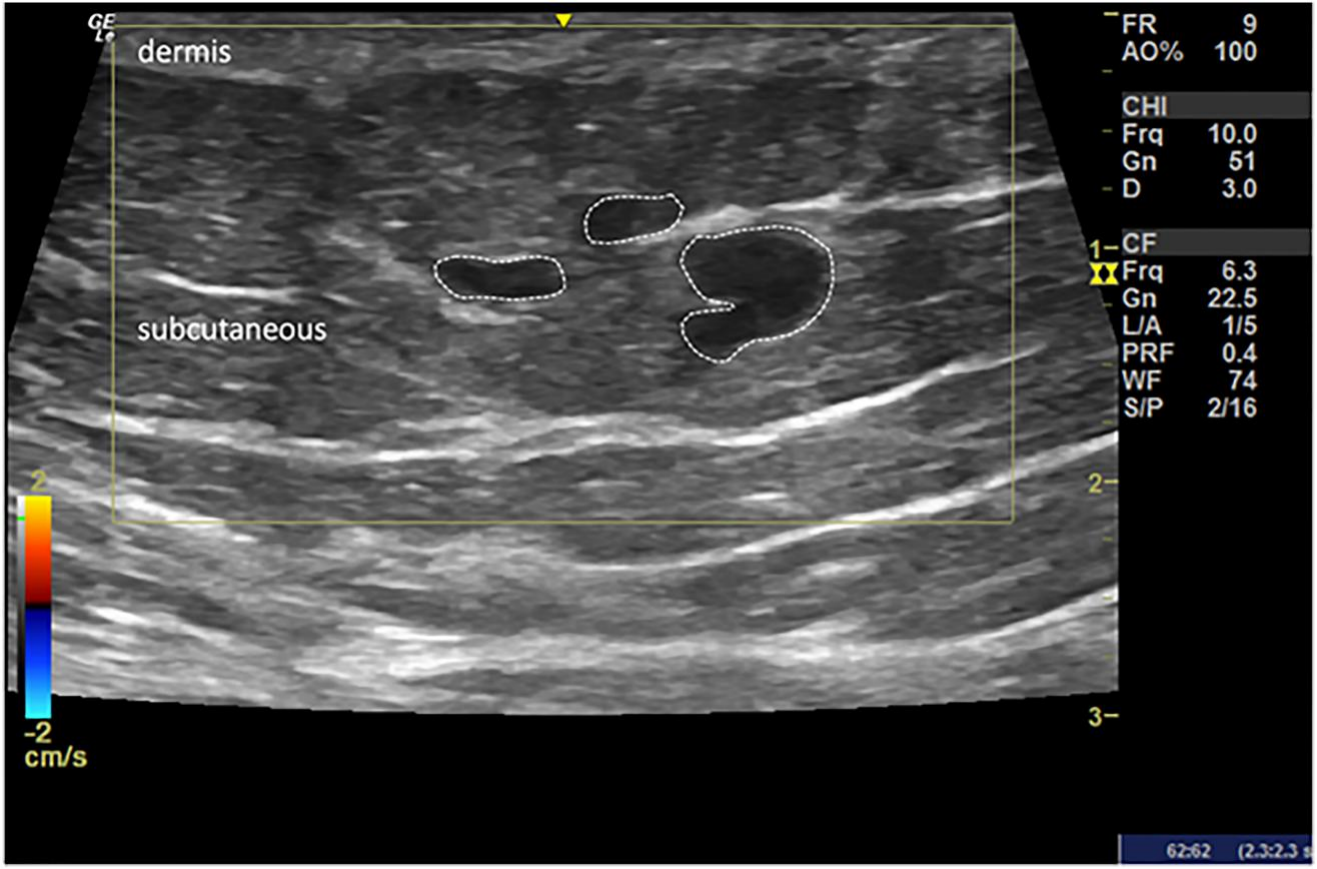
Colour Doppler ultrasound image of the plan chosen. Dotted lines represent the deposits of hyaluronic acid (HA) placed on a plan where no major vessels were found.

The plane chosen through cutaneous ultrasound for application of the product and for carrying out the movements was the subcutaneous plane, approximately 8 mm from the skin surface. The ultrasound used was the high‐resolution system Logiq e^®^ (GE Healthcare, Pittsburgh, PA) with a 10 MHz probe (Figure 1).

This plan allows anatomical safety, because there are no large vessels or important structures, unlike the muscular and submuscular region, where larger‐calibre nerves and vessels travel, such as the superior and inferior glutaeal veins. Also, this plan permits a better use of the product and allows a non‐traumatic destruction of the septa through the cannula, related to clinical lesions of cellulitis. To determine the specific site for application, the authors used the areas with the greatest number of lesions.

**FIGURE 2:**
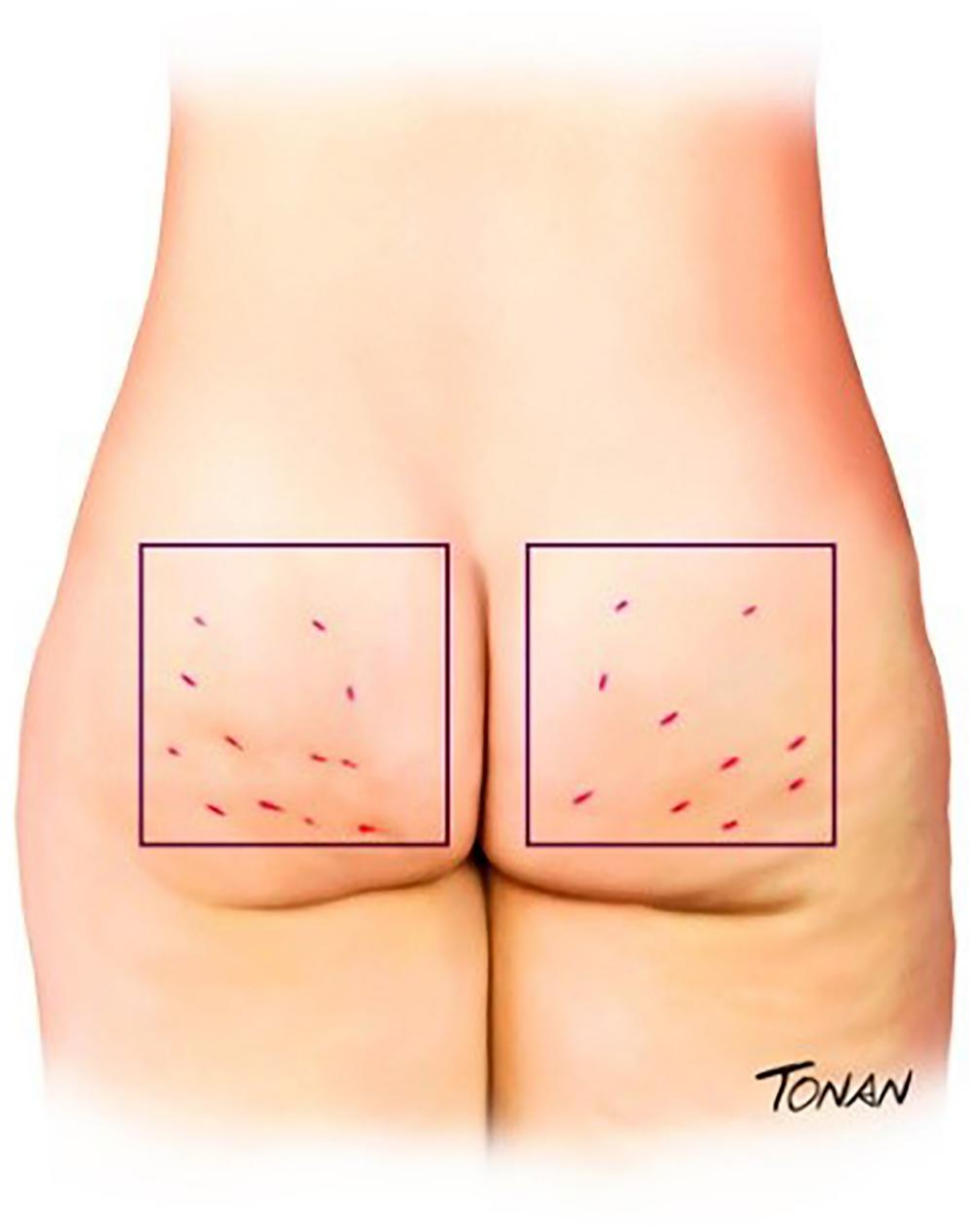
Cellulites must be demarcated.

**FIGURE 3:**
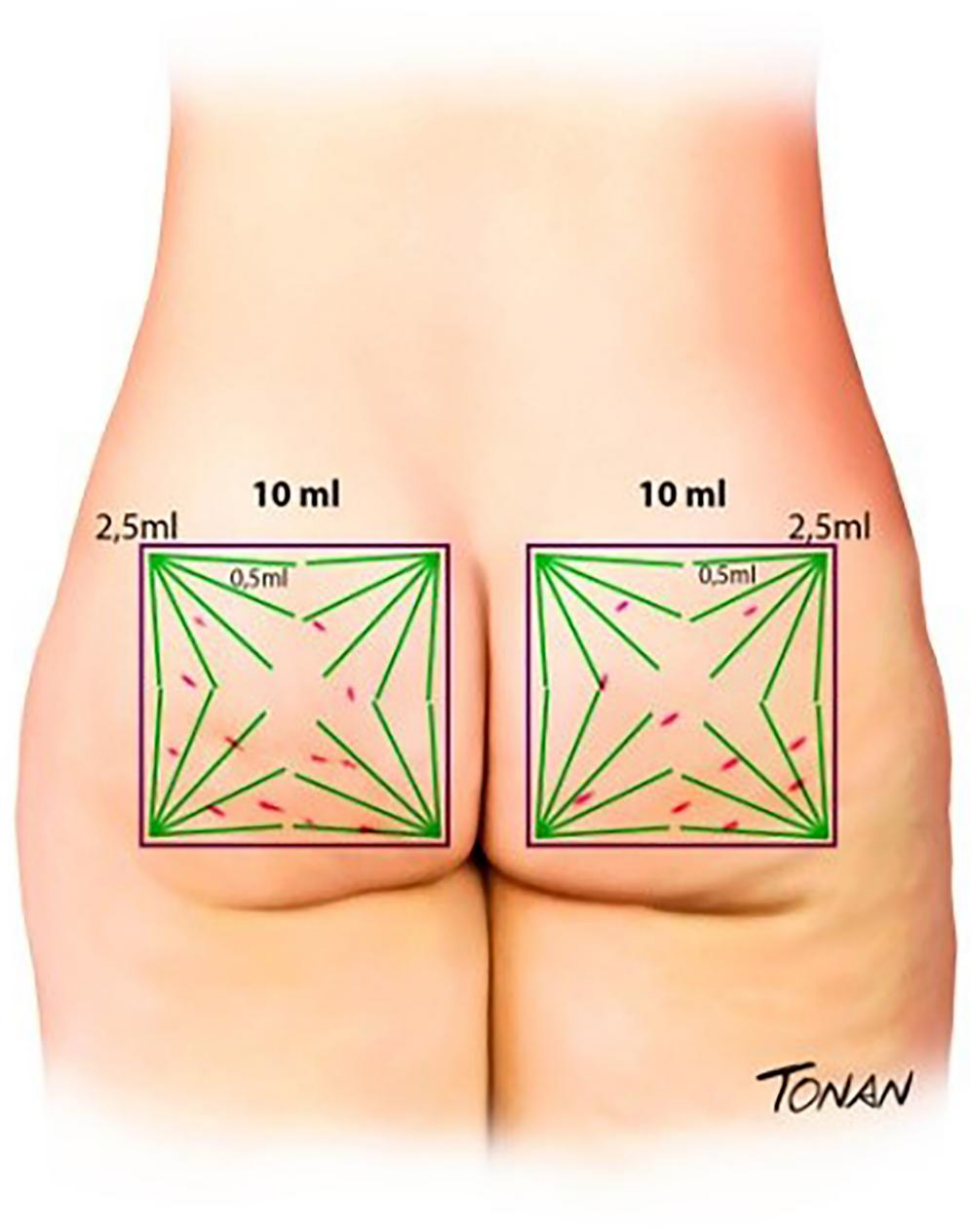
Traced square (10 × 10 cm) at the site with the greatest number of lesions. Then, five fan lines must be drawn in at all angles of the square.

The lesions must be demarcated so that the chosen site can be oriented (Figure 2). The authors traced a square (10 × 10 cm) at the site with the greatest number of lesions. Within this square five fan lines must be drawn in at all angles of the square (Figure 3).

In each line of this fan, repeated movements of entry and exit of the cannula must be made, trying to achieve a non‐traumatic cannulated treatment of the septa. These movements with the cannula would open spaces for the HA of large particles to fill the place improving the surface. Lateral movements should not be made with the cannula, only entry and exit movements to make room for the entry of HA in large particles, avoiding major trauma.

Then, the authors suggest the application, also through the same cannula, of 0.5 mL of HA in each arm of the fan, totaling 2.5 mL of product in the area related to each angle of the square, totaling 10 mL of the product per buttock and 20 mL across both buttocks per session.

This amount of product may vary depending on the size of the buttocks and the application can be repeated in 15 days if necessary.

An anaesthetic button with lidocaine must be made to create the entry hole.

A soft and compressive dressing must be made, the use of a compression belt for 15 days after application is necessary.

There may be erythema and local pain for 2–3 days. Prophylactic antibiotics are recommended due to the anatomical location. The authors suggest Azithromycin 500 mg for 3 days due to ease of dosing. It is recommended to avoid high‐impact exercise for 2–3 days and avoid sun exposure if there is bruising for 20 days.

The treated patients were submitted to a questionnaire on the degree of satisfaction and on adverse effects. Patients were followed up for an average duration of 10.8 months.

## 7 RESULTS

The authors report good aesthetic results with the proposed technique. Only three patients exhibited mild haematomas after the procedure and mild pain at the site for 2–3 days. No more serious complications were reported.

**FIGURE 4:**
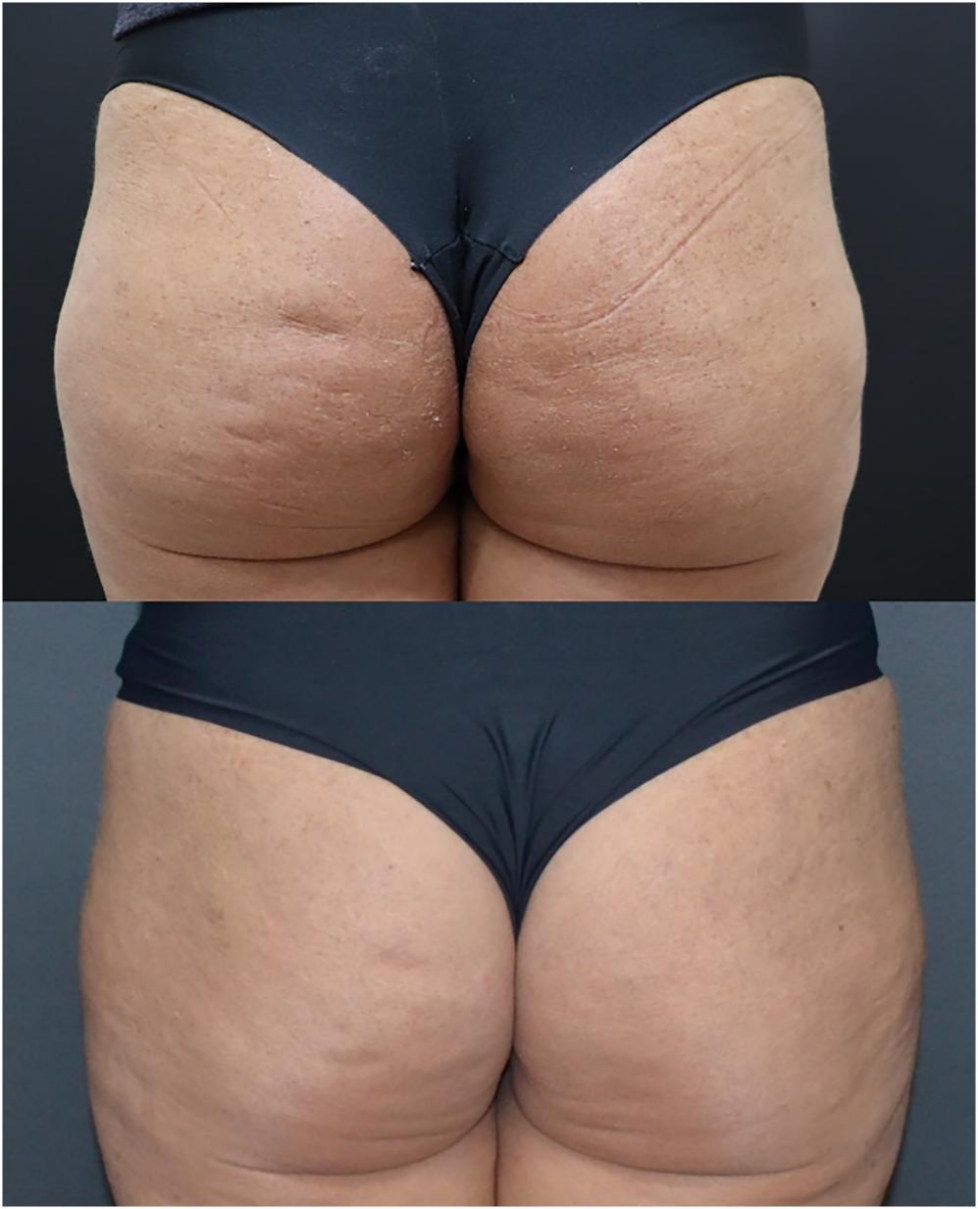
Right before the procedure and 6 months after.

**FIGURE 5:**
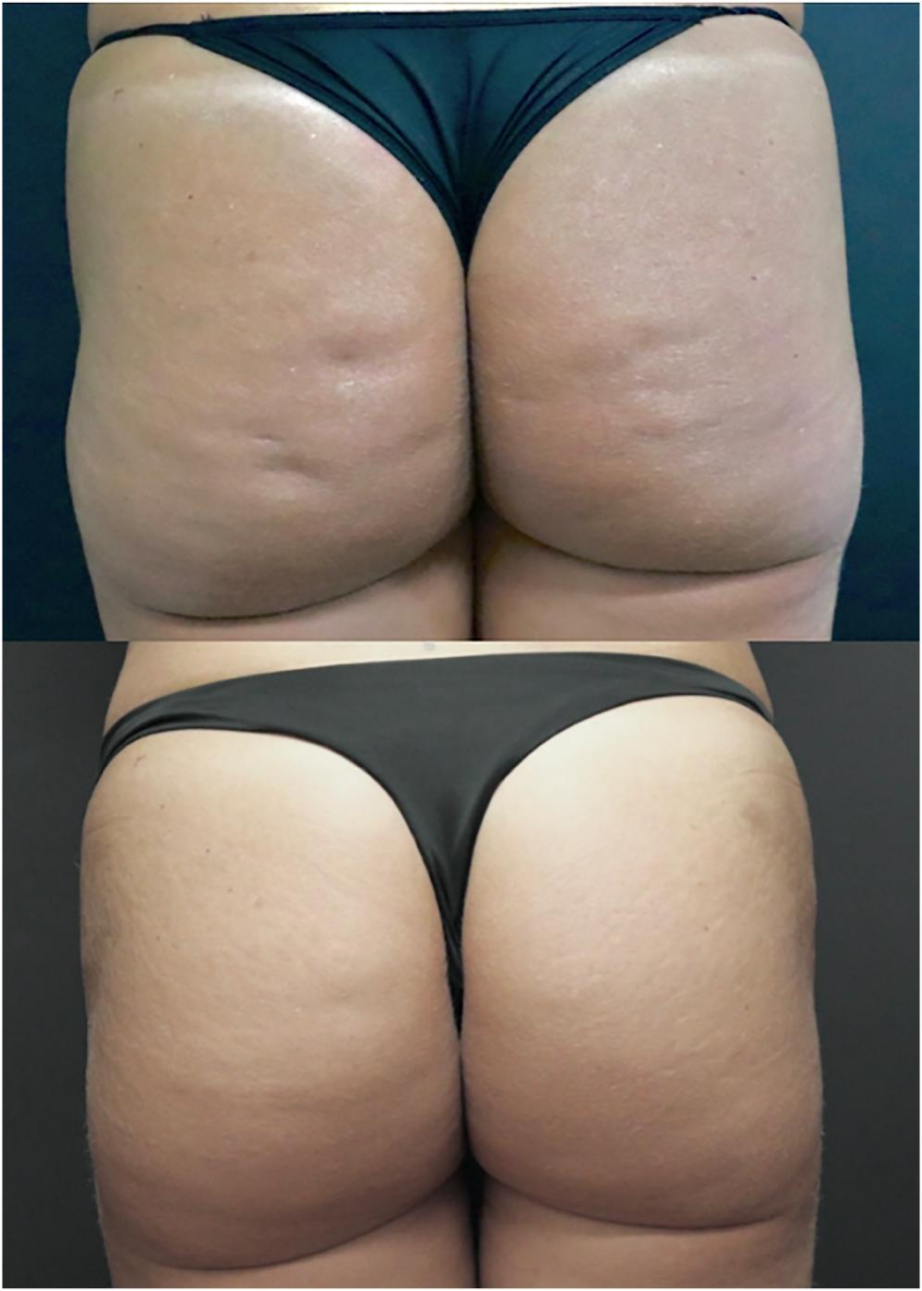
Right before the procedure and 19 months after.

It is possible to observe the improvement of cellulite lesions and improvement of the buttocks contour. During the 19 months follow up there was progressive clinical improvement (Figures 4 and 5).

Patients reported high degree of satisfaction with improvement in their quality of life.

## 8 DISCUSSION

Hyaluronic acid (HA) is a biocompatible, absorbable product that has been used in the medical field for over 25 years and is considered a very safe filler.^18^

The product chosen has high G prime and large particles, which accounts for resistance to deformation and a high lifting power.^18^ The large size of the particle (800–1800 μm) is important for body filling, so it is possible to have excellent results with less product.^18^

When it comes to body filling, where the area to be treated is larger than the face, the volume needs to be higher.^18^ In addition, having presentations of 10 and 20 mL facilitates the treatment.^18^

The subcutaneous tissue was chosen as the HA application plan in the buttocks region, due to the absence of large blood vessels or other risky structures.^19^, ^20^, ^21^, ^22^

In addition to safety, this plane allows for better use of the product, which is very important in the body region to enable the technique, since the volume of the products is greater.^19^, ^20^, ^21^

The injection in fans allows a greater spreadability of the product, which is important in an application in the subcutaneous tissue, as it makes the surface more regular, allowing a treatment of the area, important where there are several surface irregularities.

Often, when the fibrous septa present in cellulite is excised, we are unable to completely fill in the atrophic lesions of cellulite. HA applied immediately after non‐traumatic subcision^TM^ of septa by cannula allows immediate filling of the lesions in addition to a late improvement.

The patients were followed up for an average duration of 10.8 months, with progressive clinical improvement. Delayed improvement might be due to the stimulating action of HA. Some reports describe a stimulation of procollagen, growth factors in skin and induction of mesenchymal stem cell migration in the extracellular matrix secondary of the HA injection.^18^, ^23^, ^24^

In our technique, we chose to use a cannula instead of a needle, to minimise large haematomas and possible hemosiderosis and residual hyperchromia. In the technique described by the authors performs a less invasive breakage of the septa; and filling with HA applied immediately afterwards, brings excellent results in a less invasive way and with less risk of complications.

The authors reaffirm that this technique is indicated for patients with several atrophic lesions and surface irregularities but without skin flaccidity. For cellulite related to skin flaccidity, association with other techniques and/or technologies should be considered.

## 9 CONCLUSION

The Square technique combines non‐traumatic subcision^TM^ of septa by cannula and immediate filling with large particle size HA gel, for the treatment of cellulite. It proved to be a minimally invasive technique, with fast results, without major risks, little downtime, that brought satisfaction to patients with improvement in their self‐esteem and quality of life.

## CONFLICT OF INTEREST STATEMENT

The authors declare no conflicts of interest.

## AUTHOR CONTRIBUTIONS

**Luciana de Matos Lourenço**: Data curation (lead); Methodology (lead); Project administration (lead); Supervision (lead); Writing – review & editing (lead). **Lara Apendino Colla**: Data curation (equal); Writing – original draft (equal). **Maria Gabriela Ortiz de Noronha**: Data curation (equal); Writing – review & editing (equal). **Thomaz Rezende Izzo**: Data curation (equal); Writing – original draft (equal). **Rosa Sigrist**: Data curation (equal); Formal analysis (equal); Resources (equal); Writing – review & editing (equal).

## ETHICS STATEMENT

The research was conducted in accordance with the principles embodied in the Declaration of Helsinki and in accordance with local statutory requirements.

## Data Availability

The data that supports the findings of this study are available from the corresponding author upon reasonable request.
